# Notes

**Published:** 1896-04

**Authors:** 


### Notes.

---

The *St. Joseph Medical Herald* for March contains the usual half-tone engraving of the nude. The subject is the 'Birth of Venus." Venus, for a newborn child, is strikingly developed. Her luxuriant, dusky tresses sweep down to her dimpled knees, and her generous form shows symmetry of contour and a maturity that is absolutely unique in the annals of obstetrics. On either side of this central figure are water nymphs clasped in the arms of supposed mermen gathered to witness the travail of Oceana. In the background are winged figures romping in the air, and in the foreground are cherubs riding upon the back of a bridled dolphin; further forward is the dim figure of a merman blowing a conch shell. All very pretty and very likely will cause a large demand for the anaphrodisiac qualities of Peacock's Bromides, the advertisement of which with great fitness, can be found in the same volume. At the bottom of the picture, we are advised that a "New Remedy" may be seen on the other, and following this advice, we are surprised to find a prosaic description of a certain salt for lithæmia and not a word about Damiana Wafers.

If the *Medical Herald* has any difficulty in procuring these pictures, we can furnish it with the address of a dealer in Belgian *au naturel* obscenities in the Palais Royal, Paris, at the sign of the Phallus.

---

The editorial department of the *New Orleans Medical and Surgical Journal* will be conducted in the future by Drs. Charles Chassaignae and Isadore Dyer, both of whom have proven themselves eminently qualified for the task.

---

Dr. P. M. Butler has been appointed surgeon to the Seaboard Air Line.

---

The X ray may show coins in a purse, but it will never show them in a doctor's pocket.

DR. JOSEPH JONES, one of the most distinguished physicians of the South, died at New Orleans, February 18. He was the son of the Rev. Charles Colcock Jones, D. D., of Georgia. Dr. Jones devoted his life to the investigation of the causes and prevention of disease in civil and military hospitals, and was a prolific writer on the subject of his experiments. His work on yellow fever is well known.

At the time of his death, he was the incumbent of the chair of chemistry at the Tulane University, New Orleans. He married a daughter of the Confederate general and Episcopal bishop of Louisiana, Leonidas Polk.

---

THE State Medical Association will meet April 15, at Augusta. The list of papers to be read contains some very interesting titles. Dr. Goodrich, the secretary, writes that there is every indication of a full meeting. He promises a very pleasant time to the members in attendance, one of the features of entertainment being a grand barbecue. Dr. Phelps, of New York, and Dr. Howard O. Kelly, of Baltimore, will be present at the meeting and will read papers. Members of the Association are urged to be present.

---

DR. HENRY S. WRIGHT has been elected by the city council to fill the vacancy of city physician of the sixth ward, caused by the death of Dr. N. O. Harris. Dr. Wright is to be congratulated on winning the race against so many competitors.

---

BY some oversight the name of Dr. Bransford Lewis, of St. Louis, Missouri, was omitted as the author of the article on "Local Anæsthesia by the Infiltration Method" which appeared in the March number of the SOUTHERN MEDICAL RECORD.

---

THE Missouri State Board of Health is girding up its loins for work in the spring. Candidates for license in that state are reminded of the radical work of the new broom.

---

THE Practice of Medicine Act passed the Ohio State Senate, February 19. The circle is rapidly narrowing on the quacks everywhere.

